# The Effect of Visual Articulatory Information on the Neural Correlates of Non-native Speech Sound Discrimination

**DOI:** 10.3389/fnhum.2020.00025

**Published:** 2020-02-07

**Authors:** James M. A. Plumridge, Michael P. Barham, Denise L. Foley, Anna T. Ware, Gillian M. Clark, Natalia Albein-Urios, Melissa J. Hayden, Jarrad A. G. Lum

**Affiliations:** Cognitive Neuroscience Unit, School of Psychology, Deakin University, Geelong, VIC, Australia

**Keywords:** audio-visual training, speech processing, speech discrimination, mismatch negativity (MMN), event related potential (ERP), non-native speech sounds

## Abstract

Behavioral studies have shown that the ability to discriminate between non-native speech sounds improves after seeing how the sounds are articulated. This study examined the influence of visual articulatory information on the neural correlates of non-native speech sound discrimination. English speakers’ discrimination of the Hindi dental and retroflex sounds was measured using the mismatch negativity (MMN) event-related potential, before and after they completed one of three 8-min training conditions. In an audio-visual speech training condition (*n* = 14), each sound was presented with its corresponding visual articulation. In one control condition (*n* = 14), both sounds were presented with the same visual articulation, resulting in one congruent and one incongruent audio-visual pairing. In another control condition (*n* = 14), both sounds were presented with the same image of a still face. The control conditions aimed to rule out the possibility that the MMN is influenced by non-specific audio-visual pairings, or by general exposure to the dental and retroflex sounds over the course of the study. The results showed that audio-visual speech training reduced the latency of the MMN but did not affect MMN amplitude. No change in MMN amplitude or latency was observed for the two control conditions. The pattern of results suggests that a relatively short audio-visual speech training session (i.e., 8 min) may increase the speed with which the brain processes non-native speech sound contrasts. The absence of a training effect on MMN amplitude suggests a single session of audio-visual speech training does not lead to the formation of more discrete memory traces for non-native speech sounds. Longer and/or multiple sessions might be needed to influence the MMN amplitude.

## Introduction

A well-known difficulty of learning a second language in adulthood is discriminating between non-native speech sounds (Aoyama et al., 2004). Non-native speech sound discrimination can be difficult if both sounds map onto a single phoneme category for the listener (Kuhl, 2010). For example, native English speakers can have difficulty discriminating the Hindi dental (e.g., /t/) and retroflex (e.g., /ʈ/) sounds (Werker and Lalonde, 1988; Pruitt et al., 2006; MacLean and Ward, 2016). This is because English speakers perceive both the dental and retroflex sounds as mapping onto a single English phoneme (e.g., /t/) category. Because accurate phonemic discrimination is crucial to language comprehension, much work has sought to find ways to enhance the learning of non-native speech sound contrasts. For instance, a number of behavioral studies have shown that the ability to discriminate between non-native speech sounds improves after seeing how the sounds are articulated (Hardison, 2003, 2005; Hazan et al., 2005; Hirata and Kelly, 2010; Llompart and Reinisch, 2017). However, the effect this type of training has on the neural processing of non-native speech sound contrasts has received less empirical attention. The current study used electroencephalography, specifically event-related potentials (ERPs), to examine the influence of visual articulatory information on non-native speech sound discrimination.

A well-replicated finding is that audio-visual speech training improves the ability to discriminate between non-native speech sounds (Hardison, 2003, 2005; Hazan et al., 2005; Hirata and Kelly, 2010; Llompart and Reinisch, 2017). For example, Hazan et al. (2005) found that Japanese speakers’ ability to discriminate the English phonemes /b/ and /p/ from /v/, was improved by presenting each sound with its visual articulation. In a pre- and post-test design, native Japanese speaking participants were presented with the English phonemes embedded in syllables, for example, /bi/, /pi/, or /vi/. Participants were asked to select which phoneme was embedded in the syllable. As the phoneme /v/ is not used in Japanese, it was expected that the /v/ sound would map onto the Japanese /b/ phoneme category. Participants were presented with this task before and after they completed one of two training conditions. In the “audio-visual speech training” condition, participants received audio-visual presentations of words that contained the phonemes /b/, /p/ and /v/. During the training they saw that the sounds /b/ and /p/ are lip-articulated, while the /v/ sound is articulated using the lips and teeth. In a control condition, participants were presented with the same words that were used in the audio-visual speech training condition. However, they only heard the words; there was no accompanying visual articulatory information. Participants completed 10 training sessions over a 4-week period. After training, participants in the audio-visual speech training condition were more accurate in perceiving the /v/ phoneme compared to participants in the control condition. Thus, the ability to discriminate /v/ from /b/ and /p/ improved more in participants who had seen how each sound is articulated.

It is clear that the pairing of auditory and visual articulatory information facilitates the learning of non-native speech sounds (Hardison, 2003, 2005; Hazan et al., 2005; Hirata and Kelly, 2010; Llompart and Reinisch, 2017). However, it is less clear how this type of training influences non-native speech sound discrimination at the neurological level. In the current study, the influence of audio-visual speech training on the processing of non-native speech sounds was examined using the mismatch negativity (MMN; Näätänen et al., 1978) event-related potential.

The MMN is elicited using an auditory oddball paradigm in which a rare or deviant sound is presented (~20% of presentations) amongst frequently occurring or standard sounds (~80% of presentations). The MMN is a negative waveform elicited by presentation of the deviant sound (Näätänen et al., 2007). The waveform peaks approximately 150–300 ms post-stimulus onset (e.g., Aaltonen et al., 2008; Chládková et al., 2013).

The amplitude and peak latency of the MMN waveform can be used to investigate how audio-visual speech training influences the neural processing of non-native speech sound contrasts. The MMN amplitude increases as the brain becomes more sensitive to the difference between the deviant and standard (Bartha-Doering et al., 2015). For example, in the context of speech processing, the MMN amplitude is larger in magnitude when the standard and deviant are two speech sounds that map onto different phoneme categories, compared to when the sounds map onto the same phoneme category (Näätänen et al., 1997; Winkler et al., 1999; Molnar et al., 2014). One suggestion to account for the larger MMN amplitude to native speech sound contrasts is that the brain has discrete memory traces for native speech sounds (i.e., phonemic traces). These memory traces serve as recognition patterns that enhance neural sensitivity to the difference between speech sounds (Näätänen et al., 1997; Kujala and Näätänen, 2010).

The MMN amplitude appears to capture the brain’s sensitivity to non-native speech sound contrasts after training. For example, Tamminen et al. (2015) used a pre- and post-test design to test the influence of listen-and-repeat training on the MMN elicited during Finnish speakers’ discrimination of the English /f/-/v/ phoneme contrast (both sounds map onto a single phoneme /f/ in Finnish). During the training participants listened to, and repeated (i.e., produced) words containing the English phonemes. Participants completed four, 2- to 3-min training sessions over three consecutive days (two training sessions on Day 2). No feedback was given with respect to the accuracy of their productions during training. Before training, the MMN elicited by the contrast was not significant. However, after the second and fourth training sessions, a significant increase in MMN amplitude was observed. Behavioral results showed that training improved participants’ ability to categorize and discriminate the /f/ and /v/ phonemes. The increased MMN amplitude was suggested to reflect enhanced neural sensitivity to the /f/-/v/ contrast, due to the formation of more discrete memory traces for the /f/ and /v/ sounds.

The MMN peak latency reflects the time taken for the brain to process the difference between the standard and deviant (Bartha-Doering et al., 2015). This can occur independently of changes in MMN amplitude (Jakoby et al., 2011; Miglietta et al., 2013). Specifically, in the context of speech processing a reduction in latency can occur even though discrete memory traces for speech sounds have not been created. For example, Jakoby et al. (2011) compared two groups of native Hebrew speakers who differed in how well they had learned English as a second language. One group comprised highly proficient English learners. In the other group, participants had low English proficiency. The MMN was measured during participants’ discrimination of a French vowel contrast (/u/-/y/) that is not phonemic in either Hebrew or English. The results showed no group difference in MMN amplitude. However, the MMN peak latency was significantly shorter in the highly proficient group. The absence of a group difference in MMN amplitude suggests the shorter latency was not related to a difference in the discreetness (or lack thereof) of the neural memory traces for the vowels. The shorter MMN peak latency may reflect that the highly proficient group had greater sensitivity to and could therefore more efficiently process the non-native contrast. Thus, in the study by Jakoby et al. (2011), MMN peak latency revealed differences in the neural processing of a non-native speech contrast that were not captured by the MMN amplitude.

MMN latency can also be used to study the effects of training on the neural processing of non-native speech sound contrasts (Tremblay et al., 1997, 1998; Menning et al., 2002). Menning et al. (2002) used a pre- and post-test design to test whether auditory training could improve the ability of German speakers to discriminate between Japanese vowel and consonant durational contrasts. During training, participants received auditory presentations of the contrasts and were asked whether the two vowels were the same or different. Feedback was provided on whether or not the response was correct. The results showed that training lead to a significant pre- to post-test decrease in MMN peak latency by an average of 20 ms. This decrease in latency was interpreted to suggest that training increased the speed with which the brain processed the contrasts.

The MMN waveform is also sensitive to audio-visual speech. A number of studies have shown that the MMN is elicited by the McGurk illusion (e.g., Sams et al., 1991; Colin et al., 2002; Saint-Amour et al., 2007; Stekelenburg and Vroomen, 2012; Proverbio et al., 2018; Stekelenburg et al., 2018). In these studies, participants are typically presented with a congruent audio-visual standard (e.g., audio-visual syllable /ba/) and an incongruent audio-visual “McGurk” (McGurk and MacDonald, 1976) deviant. For example, a visual /va/ is dubbed over an auditory /ba/, creating the illusion of hearing /va/ (Saint-Amour et al., 2007). Presentation of the incongruent audio-visual deviant elicits the MMN, which suggests the auditory MMN waveform is sensitive to the integration of auditory and visual speech. However, in these studies the MMN was recorded during the presentation of the audio-visual stimuli. That is, it is less clear how the auditory MMN waveform is affected after audio-visual speech training.

There is some evidence suggesting that audio-visual speech training might influence the neural processing of non-native speech sound contrasts. For example, Zhang et al. (2009) found that Japanese speakers showed a larger MMN amplitude to the English /r/-/l/ sound contrast after audio-visual speech training (i.e., after seeing each sound presented with its corresponding visual articulation). The training was not found to influence MMN latency. However, the extent to which audio-visual speech training alone affected MMN amplitude is unclear. This is because Zhang et al. (2009) used additional training methods to improve discrimination of the /r/-/l/ contrast. Specifically, during training participants were presented with versions of the /r/ and /l/ sounds in which the acoustic differences between the sounds had been exaggerated. Thus, changes to the acoustic signal rather than audio-visual speech training may have influenced neural processing of the non-native speech sound contrast.

In the current study, the MMN was used to examine whether audio-visual speech training alone influences native English-speaking participants’ neural sensitivity to the Hindi dental /t/—retroflex /ʈ/ contrast. Participants were assigned to one of three training conditions. In the audio-visual speech training (hereafter referred to as “AV speech training”) condition, participants watched short videos of a female speaker producing the dental and retroflex sounds. Thus, participants saw how each sound was articulated. The two other conditions served as control conditions. In the Incongruent-Articulation control condition, participants were also shown videos of a female speaker producing the dental and retroflex sounds. However, the retroflex sound was presented with the visual articulation of the dental sound. Therefore, in this condition, the visual information could not be used to discriminate between the sounds. In the No-Articulation control condition, each sound was presented with the same still image of the speaker’s face. That is, participants in this condition did not receive any visual articulatory information. The control conditions were included in order to test whether any effect of AV speech training on the MMN was specific to participants receiving articulatory information that differentiated between the dental and retroflex sounds.

The MMN of the dental-retroflex contrast was examined before and after training. It was predicted that AV speech training would lead to a pre- to post-test increase in the MMN amplitude. It was also predicted that AV speech training would lead to a shorter MMN peak latency in the post- relative to the pre-test. In contrast, the control conditions were not predicted to influence MMN amplitude or peak latency.

## Materials and Methods

### Participants

Forty-two right-handed monolingual English-speaking adults (30 female) aged between 18 and 40 years participated in this study. Handedness was assessed using the Edinburgh Handedness Inventory (Oldfield, 1971). Participants reported no history of hearing or vision problems. Prior to testing, participants completed a Language Experience Questionnaire (see “Supplementary Materials”). The questionnaire was administered to participants to ensure that their first language was English, and also, to rule out previous exposure to the dental-retroflex contrast used in the current study. All participants provided informed consent before taking part in this study and were compensated with a $25 voucher. The study was approved by the Deakin University Human Research Ethics Committee.

Participants were pseudo-randomly allocated to one of three training conditions: the AV speech training condition, the Incongruent-Articulation condition, or the No-Articulation condition. The allocation protocol ensured the mean age and number of females and males in each condition were comparable. Fourteen participants were assigned to the AV speech training condition (*M*_age_ = 25.21 years; *SD*_age_ = 4.29 years; *n*_female_ = 8). In this condition, participants received training in which the dental and retroflex speech sounds were associated with their corresponding visual articulatory movements. Fourteen participants were allocated to the Incongruent-Articulation condition (*M*_age_ = 25.21 years; *SD*_age_ = 5.82 years; *n*_female_ = 10). In this condition, both the dental and retroflex sounds were presented with the visual articulation of the dental sound. Finally, 14 participants were assigned to the No-Articulation condition (*M*_age_ = 25.71 years; *SD*_age_ = 3.54 years; *n*_female_ = 12). In this condition, the dental and retroflex sounds were presented with a still image of the speaker’s face. No significant between-condition differences were found for gender (*F*_(2,39)_ = 1.393, *p* = 0.260, *partial* *η*^2^ = 0.067), age (*F*_(2,39)_ = 0.054, *p* = 0.948, *partial* *η*^2^ = 0.003), or handedness (*F*_(2,39)_ = 3.001, *p* = 0.061, *partial* *η*^2^ = 0.133).

### Description of Auditory and Visual Stimuli

A pre- and post-test design was used to examine the effects of AV speech training on the MMN elicited during discrimination of the dental-retroflex contrast. Participants were presented with an auditory oddball paradigm before and after completing the AV speech training or control conditions.

In the pre- and post-test oddball paradigms, a voiceless-plosive dental /t/ consonant was presented with the vowel /a/ as /ta/. This sound served as the standard. Participants were also presented with two deviant stimuli. One deviant was the voiceless-plosive retroflex /ʈ/ consonant, presented as /ʈa/. As mentioned earlier, English speakers can perceive the dental and retroflex sounds as mapping onto a single phoneme category in English (i.e., the phoneme /t/; Pruitt et al., 2006). The influence of AV speech training on the MMN elicited by the dental-retroflex contrast was the focus of this study. The second deviant was a bilabial /p/ consonant (similar to the phoneme /p/ used in English; as in the English word “pack”), presented as /pa/. We reasoned that /p/ would not be perceived as belonging to the same phoneme category as the dental /t/. This is because /t/ and /p/ phonemically contrast in English. Given the difficulty English speakers can have discriminating the dental-retroflex contrast (e.g., MacLean and Ward, 2016), the /pa/ sound was included to ensure participants were able to exhibit an MMN response in the pre-test.

Each sound had a duration of 225 ms. It is noted that the intensity of the deviants was slightly higher than the standard (retroflex, 1.4 dB; /pa/, 2.2 dB). Sound intensity can influence the MMN amplitude and latency (Jacobsen et al., 2003; Näätänen et al., 2007). However, all conditions received the same sounds throughout the entire study; the only condition differences related to the visual information participants received during training. Therefore, the effect of acoustic intensity on the MMN should be equal across all conditions.

**Figure 1:**
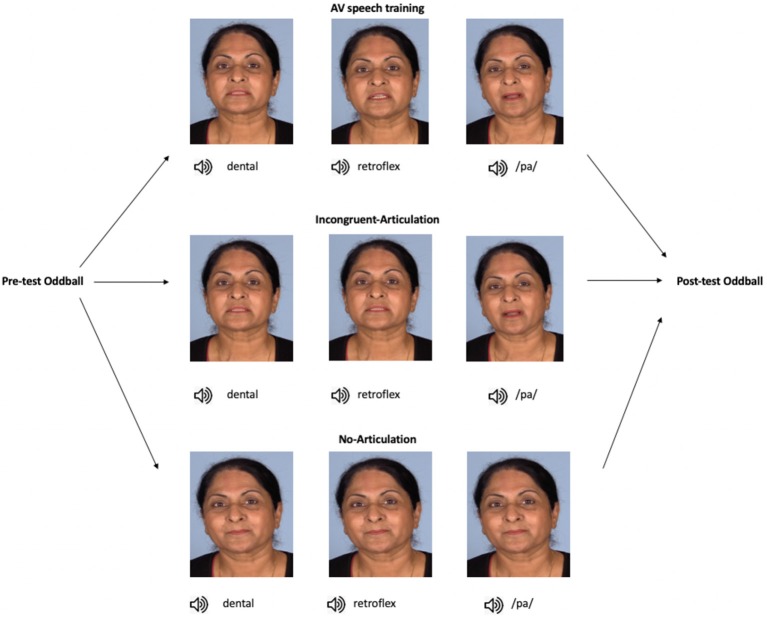
Overview of the study progression and training stimuli. In the AV speech training condition (*n* = 14), participants were presented with the dental, retroflex, and /pa/ sounds, each paired with videos of their respective visual articulation (note, the images here show stills of the different articulation of each sound). In the Incongruent-Articulation condition (*n* = 14), the dental and retroflex sounds were paired with videos of the dental articulation, and the /pa/ sound was presented with a video of its articulation. In the No-Articulation condition (*n* = 14), each sound was presented with a still image of the speaker’s face, that is, with no articulatory information. In all conditions, stimuli were presented to participants in random order. Note, informed consent was obtained from the individual shown in this figure for the publication of these images.

An overview of the stimuli and study design is presented in Figure 1. The total duration of each audio-visual presentation in all conditions was 3,000 ms, with the audio onset occurring ~1,000 ms after video onset. Participants in the AV speech training condition received individual audio-visual presentations of the dental, retroflex, and /pa/ sounds. In each presentation, only the speaker’s head was shown. The stimulus for the dental sound was an audio-visual recording of the speaker producing the dental /ta/. In this video, the tongue was seen placed between the teeth (hence dental) during articulation. In the audio-visual recording of the retroflex /ʈa/, the tongue was seen curled back in the mouth. In the audio-visual recording of the /pa/ sound, the speaker’s lips start together and open during articulation. Thus, in addition to hearing each speech sound, in each presentation participants also saw how each sound is articulated.

In the Incongruent-Articulation control condition, the audio-visual presentations of the dental and /pa/ sounds were the same as in the AV speech training condition. However, for the retroflex sound, participants saw the visual articulation of the dental sound but heard the audio of the retroflex sound (e.g., auditory /ʈa/-visual /ta/). Thus, in this condition, there was no visual articulatory information during training that differentiated between the dental and retroflex sounds. Finally, in the No-Articulation control condition participants received individual presentations of the dental, retroflex, and /pa/ sounds. In this condition, each sound was presented with the same still image of the speaker’s face with their eyes open and lips closed.

As noted earlier, the No-Articulation and Incongruent-Articulation conditions served as control conditions. There are two reasons for including these conditions. First, it could be that pairing non-native speech sounds with any visual articulation can have an effect on the MMN. If this is the case the Incongruent-Articulation condition would influence MMN amplitude and/or peak latency in a similar manner as the AV speech training condition. Second, it could be that, in the absence of any visual articulatory information, the MMN is influenced by repeated exposure to the non-native sounds over the course of the study. In this case, the No-Articulation condition would also influence MMN amplitude and/or peak latency.

To create the stimuli used in this study, a female native speaker of the Hindi language was recorded producing the dental /ta/, retroflex /ʈa/, and /pa/ sounds. The stimuli were recorded audio-visually using a video-camera (camera model: Canon XA20; video resolution: 1,920 × 1,080, 50 fps, bitrate 28,280 kbps; audio: bitrate 256 kbps, sample rate 48 kHz) in a soundproof booth. Audacity (version 2.1.0) was used to edit the auditory stimuli for the oddball paradigms. Adobe Premiere Pro (version CC 2015) was used to edit the video footage used for the training stimuli.

The soundtrack from the audio-visual recording of the dental, retroflex, and /pa/ sounds was extracted to obtain the auditory stimuli used in the pre- and post-test oddball paradigms. The audio-visual recordings also comprised the stimuli used in the AV speech training condition. As noted above, the same dental and /pa/ audio-visual recordings were used in the Incongruent-Articulation condition. To create the retroflex(audio)-dental(visual) stimulus used in the Incongruent-Articulation condition, the audio of the retroflex sound was extracted from its original audio-visual recording. This audio was then dubbed onto the visual footage of the dental sound being articulated. Finally, in the No Articulation condition the soundtrack from the dental, retroflex, and /pa/ audio-visual recordings were extracted and paired with a still image of the speaker’s face. Each sound was paired with the same still face. Thus, the pre- and post-tests, AV speech training, and control conditions all used the same audio.

### Pre- and Post-test Oddball Paradigms

The pre- and post-test oddball paradigms consisted of 1,500 trials. The standard was the dental speech sound and was presented 1,200 times (80% of trials). There were two deviants. One was the retroflex sound that was presented 150 times (10% of trials). The second deviant was the /pa/ sound that was also presented 150 times (10% of trials). The standard and deviants were presented across three blocks comprising 500 trials and presented in a pseudo-random order. The constraints placed were that the first 10 trials in each block consisted of standard stimulus presentations. Following this, the standard and deviants were randomly presented with the constraint that at least two standard stimuli preceded each deviant. The inter-stimulus interval ranged from 910 ms to 1,341 ms (*M* = 968 ms; *SD* = 7.95). The auditory stimuli were presented binaurally through headphones (Etymotic Research^®^ 30 Insert Earphones). Each test phase lasted for 30 min.

### Training Stimulus Presentation

The AV speech training and control conditions each consisted of 144 audio-visual presentations. The audio-visual presentations were organized into four 2-min blocks of stimulus presentations. There was a 1,000 ms rest between each block. Each speech sound and accompanying visual information was presented 48 times in random order. The video was presented at 29 fps using a 17″ computer monitor. The resolution of the monitor was set at 1,280 × 1,024. The audio during training was presented binaurally through the same headphones used in the pre- and post-test oddball paradigms. Each audio-visual stimulus presentation commenced with a fixation cross that appeared for 500 ms. The fixation cross was located in line with where the speaker’s mouth would appear. For the AV speech training and Incongruent-Articulation conditions, this aimed to prime the participant to fixate on the mouth of the speaker for each audio-visual stimulus presentation. The total time required to administer each training condition was 8.3 min.

### Procedure

During the study, participants were seated in a comfortable chair. All stimuli were presented to participants using E-Prime 2.0 software (Psychology Software Tools, Pittsburgh, PA, USA). Given the length of time participants would have the headphones in their ears (70 min) and the depth they were required to be inserted, before the session started participants were asked to set the volume to a level they found comfortable and not distracting. During the pre- and post-tests, participants were told to ignore the sounds being presented and watch a silent movie. Before training, participants were told to pay attention to each sound. For participants in the AV speech training and Incongruent-Articulation conditions, this instruction included watching how each sound was articulated. After training, we confirmed with participants by self-report that they had clearly heard the sounds during training.

### EEG Recording

Electroencephalography (EEG) data in the pre- and post-test oddball paradigms were recorded using a 60-channel Neuroscan AgCl electrode cap with a 10–10 electrode placement. The ground electrode was placed at AFz. Single AgCl electrodes were placed on the left and right mastoids. Bipolar referenced EOG was acquired from electrodes on the left and right outer canthi (HEOG) and also from electrodes placed above and below the left eye (VEOG). The reference electrode was placed on the nose. The EEG signal was acquired on a SynAmps RT system using Curry 7 software. Electrode impedance was measured using Curry 7 software and kept below 5 kΩ during recording. Data were acquired at a sampling rate of 1,000 Hz with a low-pass filter of 400 Hz.

### EEG Data Pre-processing

Pre-processing was undertaken using EEGLAB (version 13.6.5b; Delorme and Makeig, 2004) and ERPLAB (version 6.1.4; Lopez-Calderon and Luck, 2014) run in MATLAB (version 2016a). EEG data were first downsampled to 250 Hz. The data were then re-referenced to the mean of the two mastoids and band-pass filtered at 1–30 Hz. Stimulus locked epochs for the standard and deviant stimuli were created from the EEG data commencing −100 ms before to 600 ms after stimulus onset.

Ocular artifacts (blinks, horizontal and vertical eye-movements) and non-ocular artifacts were identified using Independent Component Analysis in EEGLAB and the EEG signal was corrected using the ADJUST algorithm (Mognon et al., 2011). MMN waveforms were then created for each deviant. First, separate averaged ERP waveforms were created for the standard and two deviants. Two sets of difference waves were then generated. In one set, the standard ERP waveform was subtracted from the retroflex deviant waveform. In the other set, the standard ERP waveform was subtracted from the /pa/ deviant waveform. This gave two contrasts: dental-retroflex and dental-/pa/.

**Figure 2:**
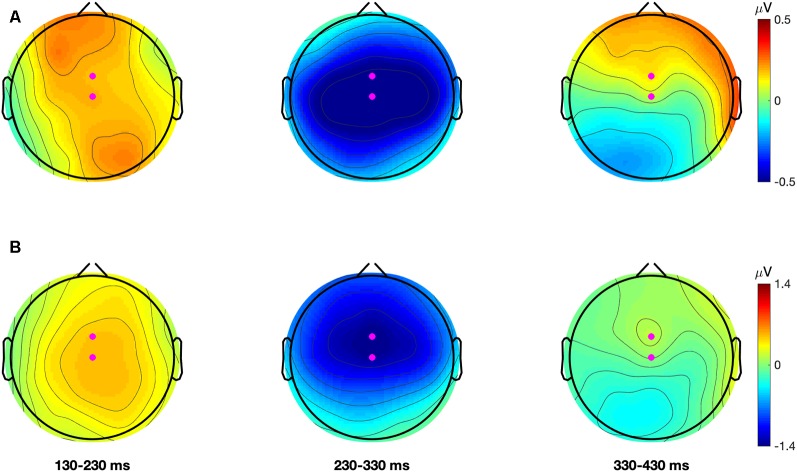
Grand average (all participants and conditions) topographical plots for the dental-retroflex (Panel **A**; top row) and dental-/pa/ (Panel **B**; bottom row) difference waves. Each plot describes the average amplitude in 100 ms time-windows. Magenta dots represent electrodes FCz and Cz.

To select the region for statistical analysis the MMN data was averaged across all participants in both test-phases. This was done separately for each contrast. In these grand-averaged waveforms, for both contrasts, the MMN showed a maximal negative peak at electrode FCz followed by Cz. Grand-average topographical plots showing the negative amplitude of the difference waveforms for each contrast are presented in Figure 2 (note also, figures of the grand-averaged waveforms at all electrodes and condition-averaged topographical plots for each contrast and test-phase can be found in Supplementary Figures S1 and S2, respectively). The finding that the MMN showed a fronto-central maximum peak is consistent with past literature (Näätänen et al., 2007). To improve the signal-to-noise ratio, the MMN data for both contrasts were averaged over FCz, FC1, FC2, Cz, C1, and C2. This created one fronto-central region of interest. Artifact rejection was then performed on this region and data points that exceeded a threshold of ±100 μV were rejected. The number of remaining trials in the AV speech training (*M*_trials_ = 1,495.93; *SD* = 13.95), Incongruent-Articulation (*M*_trials_ = 1,496.32; *SD* = 11.27) and No-Articulation (*M*_trials_ = 1,496.29; *SD* = 11.17) conditions did not significantly differ (*p* = 0.975).

### Dependent Variables

The main dependent variables in the analyses were MMN mean amplitude and peak latency for the dental-retroflex contrast. Also, as mentioned earlier, the /pa/ deviant was included to ensure participants were eliciting an MMN in the pre-test. Thus, additional dependent variables used in the analyses were the MMN amplitude and peak latency data for the dental-/pa/ contrast.

**Figure 3:**
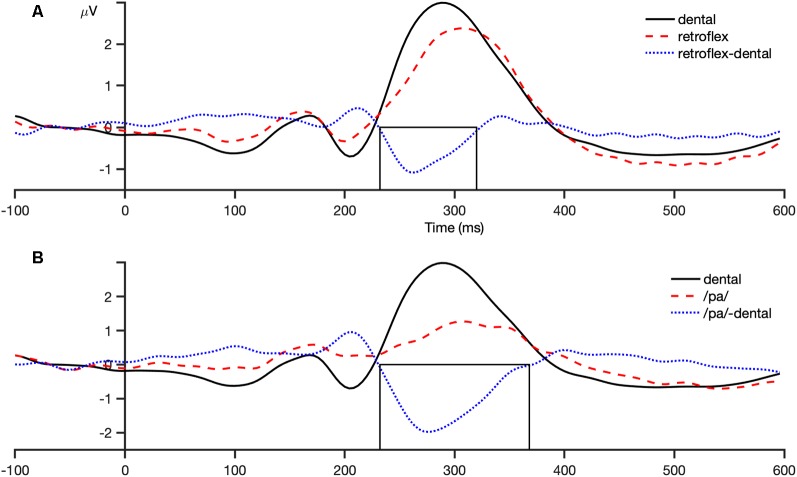
Grand average (all participants and conditions) waveforms at the fronto-central region (average of FCz, FC1, FC2, Cz, C1, C2). Panel **(A)** shows the dental-retroflex waveforms and panel **(B)** the dental-/pa/ waveforms. The boxes in each plot indicate the time-window in which each difference wave shows a negative amplitude: dental-retroflex 232–320 ms; dental-/pa/ 232–368 ms.

The analysis window for the MMN was selected by visual inspection of the grand-averaged difference waveforms of all participants and conditions for each sound contrast: dental-retroflex and dental-/pa/. A time window of 232–320 ms was selected for the dental-retroflex contrast and 232–368 ms for the dental-/pa/ contrast. These time windows corresponded to the time period in which each grand-averaged difference waveform showed a negative amplitude, that is, where subtracting the standard from the deviant yielded a negative difference wave. These waveforms are presented in Figure 3. Within these windows, for each sound contrast and test phase, the largest negative peak in the individual participant MMN waveforms was identified. Mean amplitudes were extracted from 48 ms time-windows centered at the individual peaks. The peak latency of each individual peak was also extracted.

## Results

**Figure 4:**
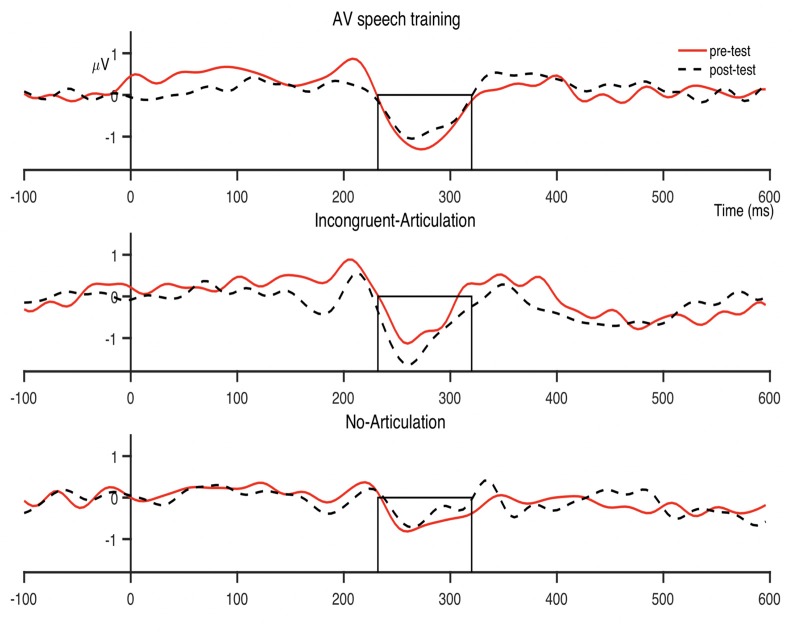
Condition averaged difference waves for the dental-retroflex contrast at the fronto-central region. The boxes indicate the analysis window within which individual participant peak amplitudes were identified (232–320 ms).

**Figure 5:**
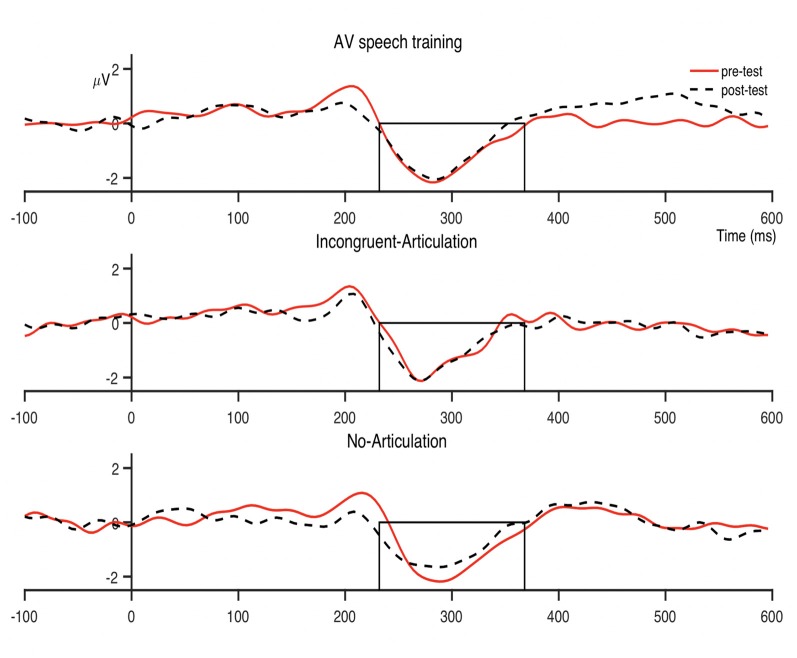
Condition averaged difference waves for the dental-/pa/ contrast at the fronto-central region. The boxes indicate the analysis window within which individual participant peak amplitudes were identified (232–368 ms).

The condition-averaged difference waveforms for the dental-retroflex and dental-/pa/ contrasts are presented in Figures 4 and 5, respectively. For all analyses, alpha was set at 0.05.

To test the hypotheses forwarded in this study, planned comparisons comprising paired samples *t*-tests were used to examine pre- to post-test changes in MMN amplitude and latency in each of the three conditions. A number of authors have recommended the use of planned comparisons to test specific hypotheses, and that such comparisons should be undertaken regardless of the outcome of omnibus tests such as ANOVA (Rosnow and Rosenthal, 1992, 1996; Furr and Rosenthal, 2003; Ruxton and Beauchamp, 2008). However, for completeness, MMN amplitude and latency data were also entered into 3 (Condition: AV speech training, Incongruent-Articulation, No-Articulation) × 2 (Time: pre-, post-test) Mixed Design ANOVAs. Holm-Bonferroni corrections were applied to the observed *p*-values in the planned comparisons for each sound contrast to control for an inflated Type 1 error rate (Holm, 1979). Effect sizes for the planned comparisons are reported using Cohen’s *d*.

### Preliminary Analysis of Difference Waves

Preliminary analyses tested whether each contrast elicited an MMN. One sample *t*-tests (two-tailed) examined whether MMN amplitudes for the dental-retroflex and dental-/pa/ contrasts in the pre- and post-tests significantly differed from a mean amplitude of zero. The MMN amplitude data and a summary of the results from this analysis are presented in Table 1. The tests revealed that the pre- and post-test MMN amplitudes for the dental-retroflex and dental-/pa/ contrasts differed significantly from zero in all conditions.

**Table 1:** Mean MMN amplitudes (μV) and one-sample *t*-test results.

		Dental-retroflex				Dental-/pa/			
Condition	Test-phase	*M*	*SD*	*t* (*df* 13)	*p*_corrected_	*M*	*SD*	*t* (*df* 13)	*p*_corrected_
AV speech training	Pre	−1.28	1.06	−4.55	0.005	−2.14	1.00	−8.02	<0.001
	Post	−1.01	1.22	−3.09	0.018	−2.02	1.24	−6.08	<0.001
Incongruent-Articulation	Pre	−1.20	1.35	−3.38	0.015	−2.00	1.44	−5.21	<0.001
	Post	−1.60	1.43	−4.20	0.005	−1.99	1.66	−4.49	0.001
No-Articulation	Pre	−1.03	0.75	−5.18	0.001	−2.09	1.23	−6.33	<0.001
	Post	−0.89	1.34	−2.50	0.027	−1.83	1.10	−6.21	<0.001

*Holm-Bonferroni correction applied to pre- and post-test p-values for each sound contrast*.

### Effects of AV Speech Training on MMN Amplitude

The first set of analyses examined the effect of AV speech training on MMN amplitude for the dental-retroflex contrast. An ANOVA revealed the Condition × Time interaction (*F*_(2,39)_ = 1.570, *p* = 0.221, *partial* *η*^2^ = 0.074) and the main effect of time (*F*_(1,39)_ = 0.001, *p* = 0.976, *partial* *η*^2^ = 0.000) were not statistically significant. Planned comparisons found no significant change in MMN amplitude for the dental-retroflex contrast in the AV speech training condition (*t*_(13)_ = −1.13, *p*_corrected_ = 0.831, *d* = 0.238). The comparisons for the Incongruent-Articulation (*t*_(13)_ = 1.76, *p*_corrected_ = 0.306, *d* = 0.285) and No-Articulation (*t*_(13)_ = −0.38, *p*_corrected_ = 0.999, *d* = 0.127) conditions were also not statistically significant.

The analysis of MMN amplitude for the dental-/pa/ contrast revealed the same pattern of results, the ANOVA (*F* < 1) and planned comparisons (*t* < 1) were not significant. Thus, overall, AV speech training was not found to influence MMN amplitude.

### Effects of AV Speech Training on MMN Latency

**Figure 6:**
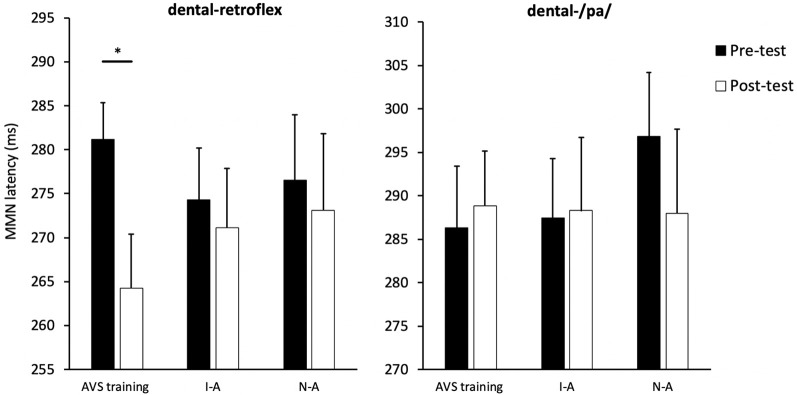
Condition averaged MMN peak latencies for each sound contrast and test-phase. AV speech training (AVS training), Incongruent-Articulation (I-A), and No-Articulation (N-A). *p < 0.05 for pre- to post-test change. Error bars show standard error.

The MMN peak latency data are presented in Figure 6. The next set of analyses examined the effect of AV speech training on MMN peak latency for the dental-retroflex contrast. An ANOVA revealed the Condition × Time interaction (*F*_(2,39)_ = 1.755, *p* = 0.186, *partial η*^2^ = 0.083) was not statistically significant. However, the main effect of time (*F*_(1,39)_ = 5.227, *p* = 0.028, *partial η*^2^ = 0.118) was significant. There was an overall decrease in latency from pre- (*M* = 277.33; *SD* = 22.05) to post-test (*M* = 269.52; *SD* = 26.74) for the dental-retroflex contrast.

The planned comparisons revealed a significant pre- to post-test decrease in latency for the dental-retroflex contrast in the AV speech training condition (*t*_(13)_ = 3.08, *p*_corrected_ = 0.027, *d* = 0.860). No significant change in latency was found for the Incongruent-Articulation (*t*_(13)_ = 0.57, *p*_corrected_ = 0.999, *d* = 0.133) and No-Articulation (*t*_(13)_ = 0.51, *p*_corrected_ = 0.999, *d* = 0.114) conditions. Finally, the results of the ANOVA (*F* < 1) and planned comparisons (*t* < 1) on MMN latency for the dental-/pa/ contrast were not significant.

### Analysis of the Influence of AV Speech Training on the Relationship Between Dental-Retroflex MMN Amplitude and Peak Latency

Exploratory analyses tested whether the shorter MMN peak latency after AV speech training was related to MMN amplitude. Pearson correlations were used to examine the relationship between post-test MMN amplitude and peak latency for the dental-retroflex contrast. The correlations between post-test MMN amplitude and peak latency for the AV speech training (*r* = −0.046, *p* = 0.875), Incongruent-Articulation (*r* = −0.033, *p* = 0.910), and No-Articulation condition (*r* = 0.033, *p* = 0.911) were all non-significant. Thus, there was no relationship between MMN amplitude and peak latency after training for any of the conditions.

## Discussion

This study investigated the effects of audio-visual speech training (referred to as AV speech training) on the brain’s sensitivity to a non-native speech sound contrast when measured using the MMN waveform. It was predicted that AV speech training would increase the MMN amplitude and decrease its peak latency in the post- relative to the pre-test. No pre- to post-test change in either MMN amplitude or peak latency was expected for the control conditions. The results partially supported the hypotheses. AV speech training led to a decrease in MMN latency but did not affect amplitude. The control conditions did not affect MMN amplitude or latency. Overall, these results suggest a single, short session of AV speech training may improve the speed with which the brain is able to process a non-native speech sound contrast.

### The Effect of AV Speech Training on MMN Amplitude

The finding that AV speech training did not influence MMN amplitude is inconsistent with some past research. As noted earlier, there is evidence suggesting that AV speech training contributes to a significant increase in MMN amplitude (Zhang et al., 2009). One reason for the absence of an effect of AV speech training on MMN amplitude could be that the training was of insufficient duration to affect MMN amplitude. The studies undertaken to date that have found training influences MMN amplitude have used substantially longer training sessions than the current study and/or multiple sessions (Menning et al., 2002; Zhang et al., 2009; Tamminen et al., 2015). For example, in the study by Zhang et al. (2009), participants completed 12 × 60-min training sessions. In the study by Tamminen et al. (2015), participants completed two training sessions comprising 2–3 min per session and also completed several behavioral tasks before and after each training session that required them to discriminate between the non-native sounds. The additional practice discriminating the sounds combined with multiple training sessions could have contributed to the increased MMN amplitude (Tamminen et al., 2015).

The MMN amplitude during speech sound discrimination is thought to be enhanced by the presence of memory traces for those sounds (Näätänen et al., 1997; Kujala and Näätänen, 2010; Tamminen et al., 2015). The absence of an MMN amplitude increase in the current study may be indicating that a single 8-min session of AV speech training was not sufficient for the brain to form new memory traces, at least for the dental and retroflex speech sounds in native English-speaking adults. However, given the limited data, this explanation is necessarily tentative. To investigate this claim more thoroughly, future research could test the amount of time and number of sessions required for AV speech training to induce the formation of new memory traces for non-native speech sounds. Such findings may have important implications for second language learning. For example, this information may help educators structure class times in order to streamline adult learning of non-native speech sound contrasts.

There was however, a non-significant pre- to post-test increase in dental-retroflex MMN amplitude for the Incongruent-Articulation condition (this increase can be seen in the Incongruent-Articulation condition waveforms presented in Figure 4). This potential increase may reflect a response to the incongruent audio-visual speech information presented to participants in this condition during training. Indeed, past research has shown the MMN amplitude increases during perception of incongruent McGurk audio-visual speech stimuli (e.g., Colin et al., 2002; Saint-Amour et al., 2007; Stekelenburg and Vroomen, 2012; Proverbio et al., 2018; Stekelenburg et al., 2018).

### The Effect of AV Speech Training on MMN Peak Latency

AV speech training was found to decrease MMN peak latency for the dental-retroflex contrast by ~17 ms. This decrease is comparable to the findings of Menning et al. (2002), who found auditory training led to a pre- to post-test MMN latency decrease of about 20 ms during German speakers’ discrimination of Japanese vowel and consonant durational contrasts. The current latency decrease suggests the pairing of auditory and visual articulatory information leads to faster neural processing of the difference between non-native speech sounds (Menning et al., 2002; Jakoby et al., 2011; Bartha-Doering et al., 2015). There was no change in MMN peak latency found in the two control groups. Therefore, presenting non-native speech sounds with a still face, or presenting the sounds with the same articulatory movement is not sufficient to impact MMN latency. Thus, the results seem to indicate that increasing the neural processing speed of non-native speech sound contrasts, at least using an 8-min training session, requires that both sounds are presented with their corresponding articulatory movements.

The non-significant differences in MMN latency and amplitude for the control conditions discounts the possibility that differences in the acoustic properties of the speech stimuli led to the pre- to post-change in the AV speech training condition. As noted in the Methods, the acoustic intensity of the retroflex sound was slightly higher (1.4 dB) than that of the dental sound. If differences in the acoustic properties of the sounds were the primary variable influencing the MMN, comparable latency and amplitude results should have been observed for all three conditions. However, this was not the case.

As noted in the introduction, it is unclear from past research (e.g., Zhang et al., 2009) whether AV speech training alone is able to influence the neural processing of non-native speech sound contrasts. The results of this study provide some evidence that addresses this gap. Specifically, a single, 8-min AV speech training session appears to increase the speed with which the brain processes non-native speech sound contrasts. Overall, the current results appear to be in line with the findings of Jakoby et al. (2011), who found that greater sensitivity to a non-native speech sound contrast was reflected in a shorter MMN peak latency and not by modulation of MMN amplitude (see also Miglietta et al., 2013).

### Limitations and Suggestions for Future Research

There are several caveats that should be considered when interpreting the results for the dental-retroflex contrast. The first is that the interaction between condition and test-phase (pre- to post-test) in the ANOVA on MMN peak latency was not statistically significant. Therefore, the effect of AV speech training on MMN latency should be interpreted with caution. This non-significant finding might relate to insufficient statistical power. A medium effect size was observed (*partial* *η*^2^ = 0.083) for the interaction. However, *post hoc* power-analysis revealed there was only a 34.5% chance of detecting this effect. Thus, poor statistical power rather than a lack of an effect may explain the results of this analysis.

To evaluate the robustness of the effect of AV speech training on MMN latency, future studies could consider using larger sample sizes. Another possibility would be to test the duration of the latency effect. For example, future work could examine whether the decrease in latency is present in a second post-test session (e.g., on a separate day). If the latency decrease is still present in a follow-up testing session, this would provide evidence that AV speech training can induce a robust change in the neural processing speed of non-native speech sound contrasts.

It would be useful for future work to investigate how the current latency findings translate to the behavioral processing of non-native speech contrasts. The results presented in this study were interpreted to suggest AV speech training may improve how efficiently the brain processes non-native speech sound contrasts. How this translates to speech processing at the behavioral level is not clear and is therefore, a limitation of this study. One possibility is that a reduction in the MMN latency following AV speech training may have led to faster reaction times for this group at the behavioral level, compared to the two control groups. Future work could address this limitation by studying the effects of AV speech training on non-native speech sound discrimination using combined behavioral and neurological measures.

Finally, only one token of each of the dental and retroflex sounds was used throughout the entire study. Therefore, it is unclear whether the effects of AV speech training on the MMN latency would generalize to untrained tokens of the dental and retroflex sounds. Examining the generalization of training effects has important implications for determining the utility of AV speech training as a way to assist second language learners. Future research will be needed to determine whether the effects of AV speech training on the MMN generalize to untrained sounds.

## Conclusion

This study provides evidence suggesting that AV speech training alone can influence the neural correlates of non-native speech sound discrimination. The effect of AV speech training on MMN peak latency for the dental-retroflex contrast may be indicating that training increases the speed with which the brain processes the difference between these sounds. However, the absence of a training effect on MMN amplitude suggests a single, 8-min AV speech training session did not lead to the formation of more discrete memory traces for the dental and retroflex sounds. Overall, the results of this study suggest that future studies should consider using multiple and/or longer AV speech training sessions combined with behavioral measures when investigating the effect of training on the neural processing of non-native speech sound contrasts.

## Data Availability Statement

The datasets generated for this study are available on request to the corresponding author.

## Ethics Statement

The studies involving human participants were reviewed and approved by Deakin University Human Research Ethics Committee. The participants provided their written informed consent to participate in this study. Written informed consent was obtained from the individual(s) for the publication of any potentially identifiable images or data included in this article.

## Author Contributions

JP designed the experiment and conducted the analyses. MH provided training on EEG acquisition. JP, MB, DF, AW and GC collected the data. JL and MH provided training on EEG analysis software. JP and JL wrote the manuscript. NA-U, MB and GC edited the manuscript.

## Conflict of Interest

The authors declare that the research was conducted in the absence of any commercial or financial relationships that could be construed as a potential conflict of interest.
