# Supplementary material for: The Effect of Visual Articulatory Information on the Neural Correlates of Non-native Speech Sound Discrimination
Source: Front Hum Neurosci. 2020 Feb 7;14:25. doi: 10.3389/fnhum.2020.00025 (PMC7019039; doi:10.3389/fnhum.2020.00025)
Supplement: Supplementary file 1 [file Data_Sheet_1.docx]

**Supplementary Materials**

**Language Experience Questions**

Is your native language (i.e., the language you would speak at home) English?

Have you ever lived in a foreign country where the official language was not English? If so, for how long?

Have you ever lived outside of Australia? If so, what country and region? How long were you there? What was the main language spoken in that region?

Are you currently studying (i.e., formally or informally) a language other than English? If so, what language, and how long have you been studying it?

Have you ever studied (i.e., formally or informally) a language other than English? If so, what language and for how long did you study it?

Are you regularly exposed to a language other than English (e.g., at work if you have colleagues who frequently speak another language)?

**
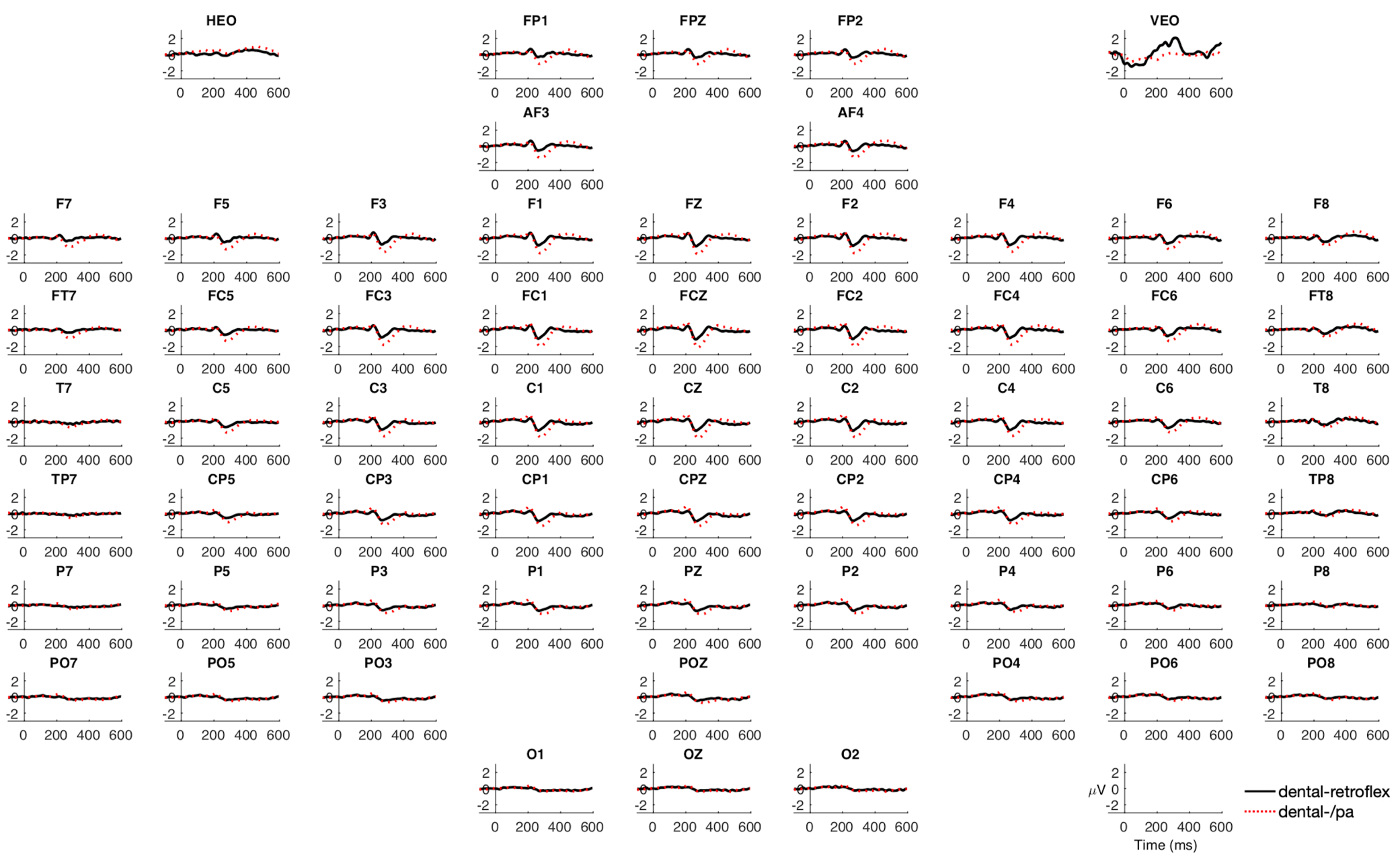
Grand-averaged waveforms at all electrodes**

**Figure S1***.* Grand-averaged dental-retroflex and dental-/pa/ waveforms at all electrodes re-referenced to the mastoids before artifact rejection. Solid lines correspond to the dental-retroflex contrast and dotted lines to the dental-/pa/ contrast.

**
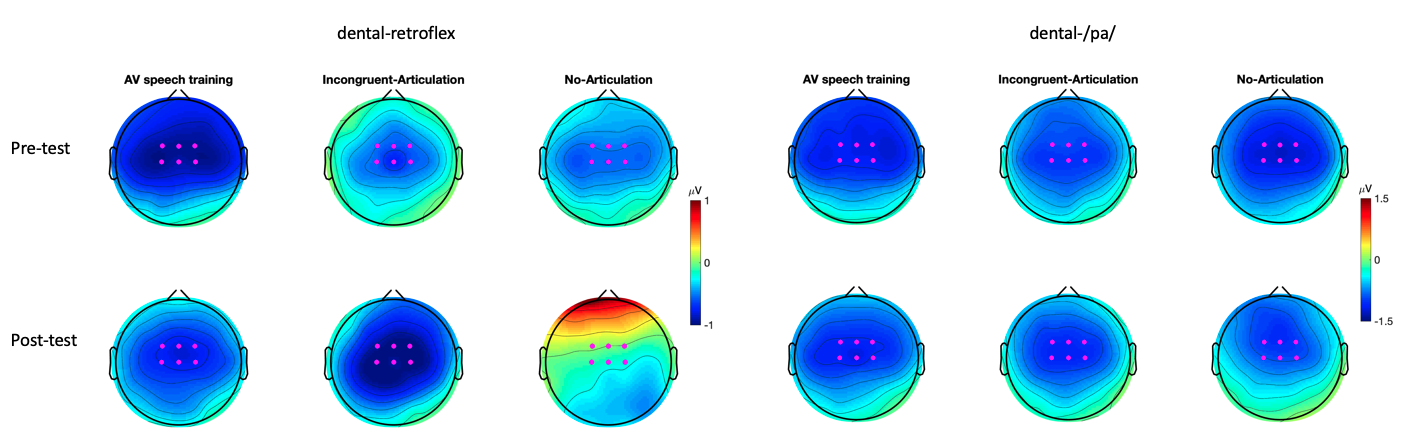
Condition-averaged topographical plots**

**Figure S2***.* Condition-averaged topographical plots for the pre-test (top-row) and post-test (bottom-row) dental-retroflex and dental-/pa/ contrasts before artifact rejection. Each plot reflects the average amplitude in the time-window used in the analysis of each contrast: dental-retroflex, 232-320ms; dental-/pa/, 232-368 ms. The magenta dots reflect electrodes used in the statistical analysis (FC1, FC2, FCZ, C1, C2, and CZ).
